# Supplementary material for: Seasonal forcing and waning immunity drive the sub-annual periodicity of the COVID-19 epidemic
Source: PLoS Pathog. 2026 Apr 27;22(4):e1014169. doi: 10.1371/journal.ppat.1014169 (PMC13138748; doi:10.1371/journal.ppat.1014169)
Supplement: S1 Table — The results of a multivariable linear regression between COVID-19 cases and all numerical variables considered (except for temperature variability and population density as they are combinations of the minimum and maximum temperatures and population size and geographic area respectively). The table includes the coefficients for each variable in the model and their 95% confidence intervals. Variables with significant coefficients are bolded. The entire model is significant with the adjusted r2 = 0.541. (PDF) [file ppat.1014169.s013.pdf]

Table 1: **Parameterization for simulations of SIR model with waning immunity.**
The three parameters that govern the waning immunity function were varied based on Latin hypercube sampling. The ranges they were chosen from are given. The other parameters were fixed across all 125,000 simulations.

Parameter	Value
R_0	2.5
$1/\gamma$	7 days
ω_∞	(0,0.75)
k	(0.5,10)
λ	(25,250)
Population size	1e6
Initial infected	1
Simulated time	3650 days
θ	0, 0.025, 0.05, 0.1, or 0.2
s_θ	0.5
