# Supplementary material for: Seasonal forcing and waning immunity drive the sub-annual periodicity of the COVID-19 epidemic
Source: PLoS Pathog. 2026 Apr 27;22(4):e1014169. doi: 10.1371/journal.ppat.1014169 (PMC13138748; doi:10.1371/journal.ppat.1014169)
Supplement: S2 Table — The three parameters that govern the waning immunity function were varied based on Latin hypercube sampling. The ranges they were chosen from are given. The other parameters were fixed across all 125,000 simulations. (PDF) [file ppat.1014169.s014.pdf]

Table 1: **Multiple regression of county-level COVID-19 cases to all climatic and demographic variables.**
The results of a multivariable linear regression between COVID-19 cases and all numerical variables considered (except for temperature variability and population density as they are combinations of the minimum and maximum temperatures and population size and geographic area respectively). The table includes the coefficients for each variable in the model and their 95% confidence intervals. Variables with significant coefficients are bolded. The entire model is significant with the adjusted $r^2 = 0.541$

Variable	Coefficient	95% Confidence Interval
**Minimum temperature**	$\mathbf{-1.29 \cdot 10^{-2}}$	$(-1.42 \cdot 10^{-2}, -1.15 \cdot 10^{-2})$
**Maximum temperature**	$\mathbf{4.85 \cdot 10^{-3}}$	$(2.17 \cdot 10^{-3}, 7.54 \cdot 10^{-3})$
Average monthly precipitation	$-5.81 \cdot 10^{-3}$	$(-1.18 \cdot 10^{-2}, 2.21 \cdot 10^{-4})$
**Log population size**	$\mathbf{6.19 \cdot 10^{-2}}$	$(4.94 \cdot 10^{-2}, 7.54 \cdot 10^{-2})$
**% Population age 65+**	**0.208**	$(9.20 \cdot 10^{-2}, 0.324)$
% **Population under 200% of the poverty line**	$\mathbf{-6.02 \cdot 10^{-2}}$	$(-0.120, -2.95 \cdot 10^{-5})$
**% Population insured**	$\mathbf{3.59 \cdot 10^{-3}}$	$(2.37 \cdot 10^{-3}, 4.81 \cdot 10^{-3})$
**Log geographic area**	$\mathbf{-6.54 \cdot 10^{-2}}$	$(-7.78 \cdot 10^{-2}, -5.30 \cdot 10^{-2})$
**Republican vote %, 2020 presidential election**	**0.248**	$(0.213, 0.284)$
Google mobility periodicity (retail)	$-1.17 \cdot 10^{-2}$	$(-6.51 \cdot 10^{-2}, 4.17 \cdot 10^{-2})$
**Google mobility periodicity (workplace)**	**−0.268**	$(-0.440, -9.67 \cdot 10^{-2})$
**Log total number of COVID-19 cases**	**0.320**	$(0.266, 0.373)$
