# Supplementary material for: Seasonal forcing and waning immunity drive the sub-annual periodicity of the COVID-19 epidemic
Source: PLoS Pathog. 2026 Apr 27;22(4):e1014169. doi: 10.1371/journal.ppat.1014169 (PMC13138748; doi:10.1371/journal.ppat.1014169)
Supplement: Attachment — Submitted filename: ReviewsFromScienceMagazine.pdf [file ppat.1014169.s015.pdf]

Review round: 1

Reviewer: 1

In this manuscript, Rubin and coauthors first analyze time series of COVID-19 cases at the US state and county levels to pick up periodicities that are present. They then determine that the magnitude of the annual period is correlated with minimum temperatures, with lower min temperatures associated with larger annual components of periodicity. Finally, they argue that the sub-annual periodicity observed in the data can be reproduced with a model that considers waning immunity, specifically, with an immunity kernel that captures a period of strong immunity, a transition period with partial immunity, and little immunity in the long term. Together, this is an interesting paper, with results that are mostly intuitive. I do think that a good number of the results could be presented more rigorously and that some additional results (particularly of model simulations that are not started near equilibrium) are needed to make the proposed explanations more convincing.

Biggest concerns:

The authors focus on understanding observed periodicities of COVID-19 during the first few years of SARS-CoV-2 being epidemic (2020-2023). However, all of the model-based analyses are run for 10 years, but only years 4-9 were used ‘to give the system time to equilibrate’ (see supplement). But the system in real life, did not have time to equilibrate. To me, it seems that all of these simulations/analyses should be done starting with early 2020 and the subsequent invasion phase - not after 4 years of time to allow for equilibration.

The other ‘elephant-in-the-room’ explanation is that the sub-annual periods that are observed are just because of the timing of certain variants of concern that spread. More convincing refutation of or wrangling with this explanation would significantly strengthen this work.

Other major comments:

To go along with this, the text ‘To provide intuition into possible mechanisms that could be driving this more unusual sustained sub-annual periodic behavior…’ That this sub-annual periodicity is sustained is not convincing with a 3-year dataset from 2020-2023. Also on p.8 ‘In general, the shape parameter… whether stable periodicity is possible’- again, is stable periodicity required? Only 2020-2023 data were analyzed.. do we really know that this sub-annual periodicity will persist?

Figure S1 and all wavelet analyses: since only 3 years of data are analyzed, it would be appropriate to add in a ‘cone of influence’ into all of the wavelet analyses. Are the annual and sub-annual periodicities that are identified within these cones of influence, given the short time series?

Figure S1: ‘Wavelets were relatively stable throughout the period of study allowing for us to simplify the analysis to use the global wavelet transformations.’ How was the stability of the wavelets determined (just by eye)? Given that global wavelet transformations were used for all the data analyses shown in the main manuscript, it seems key to me to (a) determine quantitatively whether the wavelet periods

were indeed stable, and (b) identify any temporal trends that might be apparent that simulations could attempt to reproduce during the ‘invasion phase’.

While Fig 1A shows that states with colder min temps have larger annual components of periodicity, it doesn’t show when those annual peaks in cases occur. I think it would be helpful in the evaluation of this hypothesis to include a plot that has log(case counts) by state over time (2020-203), with states listed as rows with warmest min temp at the top and going down to the coldest min temp at the bottom, so the reader could see whether those large annual peaks in cold states are from winter time large annual peaks rather than summer time large annual peaks, for example.

p.7: equations (1-5): given that what is being simulated is an individual-based model, this really cumbersome model does not need to be shown and the model can easily be described in words, with a figure showing different immunity kernels as examples.

It seems important to show how well (or poorly) the simulations recapitulate the observed COVID-19 dynamics. However, there are no plots in the main manuscript that show simulated dynamics. Supplemental Figures S7 (with seasonal forcing) and S8 (with seasonal forcing) show some of these dynamics, and I think some of these should be brought up to the main manuscript.

p. 8 ‘Perhaps most importantly…’ To me, it seems that the simulated dynamics in Fig S8 look very different from those in Fig S7, but this paragraph does not delve into the differences in great detail.

Code availability – please include github link for reproducibility.

Minor comments:

Title: Does the paper not largely argue that seasonal forcing drives the annual periodicity of the COVID-19 epidemic and that waning immunity drives the sub-annual periodicity? I suggest editing title for accuracy.

Please add line numbers for easy referencing, both to the main manuscript and to the supplement.

‘In the United State, multiple waves…’: Multiple waves sounds like >2, when Figure 1A indicates that it is mostly just 2 waves a year?

Citations of figures and figure panels from the main text could be improved upon. For example, ‘The state-level global wavelet spectra..[Fig 1].’ Refer to Fig 1A instead, e.g.

‘The maximum temperature of the country is also negatively..’ (this whole paragraph): where should we be looking to see all of these results? Table 1?

‘the marked dose-response relationship between climate variability and annual periodicity…’ Dose-response relationship? Perhaps rephrase for clarity.

It would be helpful to see a figure formatted like Figure S2 showing the wavelets for all 50 states, not just the 6 states shown in Figure S1.

The manuscript would benefit from some proof-reading – there are some sentences that have missing words or are fragmented.

Reviewer: 2

The authors address an interesting question: what drives the multi-annual cycles that have been observed for COVID19 in the USA? They present both an observational analysis using state and county-level data and a modeling analysis using an adapted SIRS model. The observational analysis reveals a correlation with temperature at the state-level and a less convincing correlation at the county-level. The statistical analysis is a little weaker and is missing key possible drivers such as vaccination and booster coverage.

I like the modeling analysis but I think for this to present a useful advance in terms of thinking through patterns of COVID19 going forward the authors need to tackle whether waning immunity or antigenic evolution is the mechanistic driver. Given the model is never actually fit to data I do have some concerns as to whether this is providing any more value than basic intuition. I think a genomic analysis that considers whether evolution is occurring at a rate that complements the model findings would be nice addition to this manuscript.

Major

1) (Observational analysis) I think the use of wavelets is a nice idea to tackle the multiple waves. I think Fig. 1A is fairly striking, however, I don't find Fig. 1B/C (the county-level analysis) as convincing. There appears to be a lot of hetereogeneity at the county-level that is not driven by climate. For instance, why is there lower annual periodicity in the north west? What is it about the northwest that means we should expect similar periodicity to the south east (aren't these regions "opposite" climate-wise?).

a. Isn't the value of wavelet analysis that the power spectra vary over time? But the authors aren't using the temporal component in this analysis, so how does this differ from using a fourier/ lomb-scargle periodogram? Can you use the temporal component to say something more nuanced about how the periodicity has evolved and what the drivers might be?

b. Why didn't the authors consider specific humidity, given this is supported as a key driver of respiratory pathogen transmission (e.g. Shaman 2009,2010)?

c. The regression analysis (Table S1) is limited e.g. it's strange to control for min and max temperature in the same regression model as they are so strongly correlated. I think the authors need to show that the estimated effect of temperature is robust to different specifications and controls.

d. The authors need to include climate data for Alaska and Hawaii etc. There are so many gridded global climate datasets out there that ignoring certain locations

because you didn’t download the right climate data comes across as careless (ERA5, for example, is the gold standard).

2) (Model) I like the innovation in using the more sensitive formulation of waning/ partial immunity than might be provided by a basic SIRS model. Can the authors hint at any mechanistic reason, specific to COVID19, to using their model for this question versus a standard SIRS? There is some basic critique of SIRS on page 6 paragraph 2, but why does the SIRS, in not considering “partial or waning immunity but absolute immunity” matter for modeling COVID19?

a. I think the author could do more to tie the modeling results from Fig 2 to the observations in Fig 1. Great that the model finds two outbreaks a year, but how does the model capture the seasonal gradient observed in Fig 1 i.e. why do northern locations have a stronger annual periodicity?

b. In general I felt the modeling results could be more clearly compared with the observational findings.

Vaccination is completely missing from both the model and observational analysis. Surely vaccination will determine spatial heterogeneity in outbreak patterns? I think the authors need to consider this much more closely, including vaccination rates and boosters as possible determinants in their statistical model. I also think the authors need to consider vaccination in the mechanistic disease model – can we rule out vaccination as a determinant of seasonality?

I think the authors could have done a better job at more deeply considering pathogen evolution. The authors attempt to discuss this broadly on page 8/9 but I think a quantitative analysis would have been more satisfying. It seems the authors are implying that the waning immunity in their model is not driven by evolution (paragraph 3 page 8) but could be driven by evolution (paragraph 2 page 9). Given the rich availability of strain data for SARS-CoV-2, and existing secondary analyses, can the authors more firmly rule out whether evolution is happening at a rate that could drive these sub-annual periodicities. Does it make sense that natural immunity wanes so quickly otherwise?

Minor

1) Please use line numbers – it helps with review.

2) The first paragraph is largely uncited which seems a little strange. I think some of these sentences should be supported by the literature? Is there not a review paper on seasonality and respiratory pathogens that could support “agents that transmit via the respiratory route are typically associated with a peak in the winter months”. I quite like Moriyama 2020 (ref below).

3) Paragraph 2 seems unfinished. I wanted another sentence “However, as susceptibility declines, it might expected that…”

4) Paragraph 3 – I thought the two peaks a year has been particularly clear over the last two years as opposed to in the early pandemic (but I might be wrong). Can we say anything on how this seasonality appears to have evolved over time? Also, might be helpful to show the time series in the US from 2019 to the present in the supplement.

5) Why minimum temperature? Can you show the correlation with max and mean too. Is it less strong? Is there some mechanistic reason to focus on minimum temperature?

6) Fig 1: - sub-labels are not in plot (i.e. A/B/C). Would also be helpful to refer to subfigures in the text (i.e. which part of Fig1 should I be looking at ?).

a. Include a color bar on Fig. 1A (what does purple mean?)

b. Caption should specify what the line is in B), what is the significance test, what are the p-values.

7) Page 7, last paragraph, missing parenthesis.

References

Moriyama, Miyu, Walter J. Hugentobler, and Akiko Iwasaki. "Seasonality of respiratory viral infections." Annual review of virology 7.1 (2020): 83-101

Reviewer: 3

I really liked the topic of this paper. We're going to put a lot of effort into understanding SARS-CoV-2 seasonality over the next 5 years, and this contribution is a great start. Comparing the different seasonal drivers of influenza and SARS-CoV-2 seasonality is also going to be interesting, and the authors touch on this topic in their manuscript. The question is great, the approach is great, but I think the authors know that the data aren't there yet to answer this question. For now, I would submit this as an exploratory analysis to a traditional epidemiology journals. In order of importance, my comments, critiques, and suggestions are below:

1. The SARS-CoV-2 incidence time series is simply too short to answer questions on COVID seasonality. 38 months and five peaks isn't enough, especially since we know many of the reasons for the occurrences of these individual peaks. Wave 1 ended because of the lockdowns in March-April 2020 (not because of any seasonal or waning factors). Wave 2 ended in Jan/Feb 2021 probably due to a combination of factors (lockdowns, fear) but definitely not because of population immunity. Wave 1 began because a new virus was introduced. Wave 2 began probably due to the seasonal and crowding factors mentioned in the paper. Wave 3 began becase the Delta variant arrived (not because immunity had waned). Wave 4 began because the somewhat immune-escaped and more trasmissible Omicron variant arrived (it would be very difficult to make the case for waned immunity here, but perhaps you could make the case for seasonal factors). I don't know what caused the summer 2022 wave (wave 5) and maybe this is the first wave where your analysis can begin. For

waves 1 through 4, the other causal factors are simply too strong and you have no way of estimating the effects of seasonality or waning immunity for these four waves.

2. "Little long-term immunity" and "immunity wan[ing] relatively sharply" (bottom p7) are indeed consistent with sustained sub-annual periodicity. Very good point and it needs to be made. However, this is not consistent with the COVID burden and COVID epidemics we have seen the past two winters. The mild COVID epidemics of winter 2022-2023 and 2023-2024 suggest that immunity is in fact a bit longer than we initially thought, probably lambda > 700 days and not lambda < 200 days. References 21-23 are not very good references to support the lack of long-term immunity you are talking about. The Goldberg analysis is a very rich data set and an excellent analysis, but it only compares inside the range of 6-8 months of immunity to 0-2 months of immunity; you need to show that 10-14 months of immunity and 20-24 months of immunity is the same as 6-8 months, but these data don't exist (to my knowledge, I may not be up to date). The Hernandez-Suarez paper has better data, out to 14 months. But 14 months of 'some immmunity' in this paper cannot be used to justify a lamdba value <250 where almost all immunity is lost in 8 months. The Townsend PNAS paper gets has a pretty unusual approach to this question, but even if we take the results on face value, their results show 5% chance of reinfection 144 days after natural infection.

[there's an additional problem with all of these duration of immunity studies, in that they were done during wildly changing epidemic dynamics; a study run in late 2021 would infer very short immunity because of the size of the delta and omicron waves, but a study run in 2022 would support longer-term immunity because of the low-level of infections during calendar-year 2022; this is not the authors' fault but does need to be considered when understanding the duration of immunity]

3. Wavelet approaches result in a lot of overfitting to short-term epidemic time series. My guess is that the authors are aware of this. The Grenfell paper (ref 6) has 40+ years of data and a clear non-stationary pattern, but if you use a wavelet approach on 38 months of data, a wavelet basis function can easily shift its amplitude to fit a large peak -- its period will change as well when this happens -- and this can result in a particular periodicity being inferred. Also, what are the black lines in Figure S1? The regions of high confidence?

4. Figures 3 and S9 are really great, and I was surprised you could get conclusions in some of these analyses. This is an excellent diagnostic approach to looking for something rare in epidemiology (here, sub-annual periodicity).

5. Bottom of page 7, after you introduce all the parameter detail, it feels like you should be able to fit your model to the data here with a likelihood approach.

6. Authors say "here we show short-term immunity that wanes quickly is a sufficient condition for

regular sub-annual periodicity, independent of multi-strain dynamics". Yes, true. Thieme and Yang (2002, Math Biosci 180:207) is the basis for this.

7. There must be some evidence in the sub-tropical and tropical influenza literature on sub-annual periodicity. Hong Kong has 20+ years of data and biannual peaks. Some older papers (Deyle PNAS 2016, Tamerius 2013 PLoS Pathog) have good summaries of this, but I don't know if they explicitly look at sub-annual periodicities.

8. For influenza virus, we know a little bit more about environmental drivers than you let on. The best mechanistic descriptions are in Wan Yang's papers on droplet size and the U-shaped absolute humidity relationship for influenza transmission.

9. It is plausible that the northern states have more pronounced winter peaks; this is also true in flu data.

10. I wouldn't use "tropical climates" in your ms; maybe "counties closer to sub-tropical zones".

11. The winter peak being close to the holiday period: there is no way to determine if this is a coincidence or not. Flu peaks are only sometimes close to the holiday period.

12. I don't understand what the dose-response relationship is on page 5.

13. Introduction, para 1: COVID waves can also be caused by varying patterns of response to lockdowns and the arrival of new variants.
