# Supplementary material for: Seasonal forcing and waning immunity drive the sub-annual periodicity of the COVID-19 epidemic
Source: PLoS Pathog. 2026 Apr 27;22(4):e1014169. doi: 10.1371/journal.ppat.1014169 (PMC13138748; doi:10.1371/journal.ppat.1014169)
Supplement: Attachment — Submitted filename: Response_to_reviewers.docx [file ppat.1014169.s017.docx]

Changes made based on the request of the “Journal Requirements” section of the email responding to our PLoS Pathogens Submission

1) We are happy to upload the LaTeX source for the manuscript in addition to the pdf version.
2) An Author Summary has been added.
3) The paper has been reformatted to match the Introduction, Results, Dicsussion, Conclusion format required by PLoS Pathogens.
4) We are happy to convert the figures into tif and eps format and will upload those along with the LaTeX source.
5) The supplementary material has been split off into a separate file as requested.
6) The base maps were drawn using the U.S. Census Bureau’s TIGER/Line Shapefiles, which are free to use. We have added the relevant citation to both Figure 2 and S5 and to the relevant section in Materials and Methods.
7) The wording of the funding statement has been edited to appear in format required.
8) A Competing Interests statement was already included below our statement of funding.

Our responses to and a list of the changes made based on the original Science reviews is included below

**Reviewer #1**

*The authors focus on understanding observed periodicities of COVID-19 during the first few years of SARS-CoV-2 being epidemic (2020-2023). However, all of the model-based analyses are run for 10 years, but only years 4-9 were used ‘to give the system time to equilibrate’ (see supplement). But the system in real life, did not have time to equilibrate. To me, it seems that all of these simulations/analyses should be done starting with early 2020 and the subsequent invasion phase - not after 4 years of time to allow for equilibration.*

We are pleased that Reviewer #1 found our paper interesting and intuitive. Their main concern with our work was that our model-based results focused on “equilibrium” solutions and 10-year simulation runs, rather than comparing a similar timescale to the case data we analyzed in the other sections (approximately four years). While we still feel that near-equilibrium and 10-year simulations make sense to isolate the effect of waning immunity from short-term stochastic and chaotic dynamics, we appreciate their concern. We have added the same analysis to the supplement with only the first four years of the simulation analyzed and show the change in timescale has no effect on the result. We hope that including these simulations will alleviate any concerns and help connect the simulation-based results to the real-world data-based analysis.

*The other ‘elephant-in-the-room’ explanation is that the sub-annual periods that are observed are just because of the timing of certain variants of concern that spread. More convincing refutation of or wrangling with this explanation would significantly strengthen this work.*

Their second major concern was that we did not discuss how the appearance and timing of SARS-CoV-2 variants of concern (VoCs) could explain the periodic waves we observe. Similar concerns were shared by the other two reviewers, that our analysis effectively ignored the role of viral evolution over the period in question. We by no means wish to dismiss the importance of evolution in determining how the COVID-19 epidemic played out – indeed the waves associated with the VoCs were a significant motivation for this work. It has been widely assumed that waves have been primarily driven by VoCs, yet this does not mean that other periodic mechanisms that are known to play large roles in similar diseases (e.g., waning immunity and seasonal forcing) are unimportant. We presented this work not to dismiss the importance of evolution but to investigate whether other mechanisms could have played a significant role in the dynamics of the pandemic. Our choice of wavelet analysis was motivated by that method’s relative insensitivity to the amplitude of waves, so making it less sensitive to large surges associated with VoCs such as BA.1 . However, from the comments of all reviewers it is clear that the discussion of the role of evolution needs to be improved, and we have done so. We added a much more explicit discussion of evolution of VOCs throughout the paper, including in the introduction as an explanation of how evolution was a partial motivation for this work.

Other major comments:

- *To go along with this, the text ‘To provide intuition into possible mechanisms that could be driving this more unusual sustained sub-annual periodic behavior…’ That this sub-annual periodicity is sustained is not convincing with a 3-year dataset from 2020-2023. Also on p.8 ‘In general, the shape parameter… whether stable periodicity is possible’- again, is stable periodicity required? Only 2020-2023 data were analyzed.. do we really know that this sub-annual periodicity will persist?*

We added shorter duration simulations as a more analogous comparison to 3-4 year data we analyze.

- *Figure S1 and all wavelet analyses: since only 3 years of data are analyzed, it would be appropriate to add in a ‘cone of influence’ into all of the wavelet analyses. Are the annual and sub-annual periodicities that are identified within these cones of influence, given the short time series?*

Cones of influence were added to all wavelet diagrams, and the periodicities we discuss are within them.

- *Figure S1: ‘Wavelets were relatively stable throughout the period of study allowing for us to simplify the analysis to use the global wavelet transformations.’ How was the stability of the wavelets determined (just by eye)? Given that global wavelet transformations were used for all the data analyses shown in the main manuscript, it seems key to me to (a) determine quantitatively whether the wavelet periods were indeed stable, and (b) identify any temporal trends that might be apparent that simulations could attempt to reproduce during the ‘invasion phase’.*

Including a wavelet diagram and more discussion of the temporal dynamics of the wavelets is a good suggestion. We added a figure with an example state wavelet and an explicit discussion of the stability of the periodicity during the pandemic period. We feel that this stability is surprising and an important observation and are glad to highlight it based on this feedback.

- *While Fig 1A shows that states with colder min temps have larger annual components of periodicity, it doesn’t show when those annual peaks in cases occur. I think it would be helpful in the evaluation of this hypothesis to include a plot that has log(case counts) by state over time (2020-203), with states listed as rows with warmest min temp at the top and going down to the coldest min temp at the bottom, so the reader could see whether those large annual peaks in cold states are from winter time large annual peaks rather than summer time large annual peaks, for example.*

The case time series was added to the new figure 1. We would also like to point out that we included a histogram of the timing of wave peaks in the supplement (figure S3).

- *p.7: equations (1-5): given that what is being simulated is an individual-based model, this really cumbersome model does not need to be shown and the model can easily be described in words, with a figure showing different immunity kernels as examples.*

We respectfully disagree with the suggestion to remove the model equations and focus on the individual-based algorithm. While it is true that we simulated an individual-based version of the PDE model we describe, we feel the PDE equations are clearer and more succinct to most modelers than the necessarily cumbersome and likely imprecise description of an individual-based algorithm. A short description of the individual-based algorithm is included in the supplement, and the GitHub repository is also linked for those who are interested.

- *It seems important to show how well (or poorly) the simulations recapitulate the observed COVID-19 dynamics. However, there are no plots in the main manuscript that show simulated dynamics. Supplemental Figures S7 (with seasonal forcing) and S8 (with seasonal forcing) show some of these dynamics, and I think some of these should be brought up to the main manuscript.*

While we appreciate that the reviewer liked our simulation results plots, we feel that bringing these figures into the main text would distract from our main discussion. This model has very complicated dynamics and we welcome a more detailed investigation of them. We instead chose to highlight the results as a general case study of what parameter regimes result in different forms of periodicity, rather than the simulated dynamics themselves. However, we do appreciate this concern and added a short discussion of what the simulated dynamics look like.

- *p. 8 ‘Perhaps most importantly…’ To me, it seems that the simulated dynamics in Fig S8 look very different from those in Fig S7, but this paragraph does not delve into the differences in great detail.*

Just as above, we added a short discussion of the simulation dynamics.

- *Code availability – please include github link for reproducibility.*

A link to the GitHub was already included in the “Data and methods availability” section at the very end of the main text.

Minor comments:

- *Title: Does the paper not largely argue that seasonal forcing drives the annual periodicity of the COVID-19 epidemic and that waning immunity drives the subannual periodicity? I suggest editing title for accuracy.*

We respectfully disagree with this suggestion and prefer to leave our title about how waning immunity and seasonal forcing interact to together explain the perhaps unusual sub-annual periodic dynamics.

- *Please add line numbers for easy referencing, both to the main manuscript and to the supplement.*

We are sorry the manuscript was difficult to review without line numbers. Our interpretation is that the Science magazine LaTeX guidelines do not allow for line numbers as part of the submission. We are happy to add them if that is not the case.

- *‘In the United State, multiple waves…’: Multiple waves sounds like >2, when Figure 1A indicates that it is mostly just 2 waves a year?*

Multiple means 2 or more. We left this purposefully imprecise as many regions and years experienced 3 or 4 waves in a single year.

- *Citations of figures and figure panels from the main text could be improved upon. For example, ‘The state-level global wavelet spectra..[Fig 1].’ Refer to Fig 1A instead, e.g.*

Yes, we are sorry the sub-panel letters were not included. We have remedied this in the figures and references to them.

- *‘The maximum temperature of the country is also negatively..’ (this whole paragraph): where should we be looking to see all of these results? Table 1?*

We added a reference to table 1.

- *‘the marked dose-response relationship between climate variability and annual periodicity…’ Dose-response relationship? Perhaps rephrase for clarity.*

Sorry for the confusion. We updated the language to convey our meaning more clearly.

- *It would be helpful to see a figure formatted like Figure S2 showing the wavelets for all 50 states, not just the 6 states shown in Figure S1.*

We are glad you would like to see the wavelets for all states, but it is impactable to include all 51 (50 states plus Puerto Rico) wavelets, even in the Supplement. The GitHub repository is linked to generate these figures or if allowed, we are happy to include a link to an online supplement with the figures, though they are quite large files.

- *The manuscript would benefit from some proof-reading – there are some sentences that have missing words or are fragmented.*

We are sorry for the typos and have corrected the few we found.

**Reviewer #2**

*The authors address an interesting question: what drives the multi-annual cycles that have been observed for COVID19 in the USA? They present both an observational analysis using state and county-level data and a modeling analysis using an adapted SIRS model. The observational analysis reveals a correlation with temperature at the state-level and a less convincing correlation at the county-level. The statistical analysis is a little weaker and is missing key possible drivers such as vaccination and booster coverage.*

*I like the modeling analysis but I think for this to present a useful advance in terms of thinking through patterns of COVID19 going forward the authors need to tackle whether waning immunity or antigenic evolution is the mechanistic driver. Given the model is never actually fit to data I do have some concerns as to whether this is providing any more value than basic intuition. I think a genomic analysis that considers whether evolution is occurring at a rate that complements the model findings would be ni*c*e addition to this manuscript.*

We are glad reviewer 2 found our premise interesting and liked the modeling analysis. We disagree however that the model should be fit to data. This compartment-style model is relatively simple (despite producing anything but simple dynamics) and is intended as a means to simulate different scenarios and capture the key determinants of observed dynamics rather than provide precise forecasts. While we could likely get decent fits if we tried, we feel fitting a 7-parameter model to such a complex epidemiological dataset is unlikely to provide additional insight. We also agree that a follow-up genomic analysis of timing and fitness of the different VoCs is a fantastic idea but is outside the scope of this paper. Similarly, including more variables in the analysis could be interesting, but we do not feel that it would have much impact on our findings (that the periodicity is stable and correlated to temperature). We would also like to point out that while we don’t explicitly consider vaccination, we agree it is important. Vaccination is good at protecting from severe outcomes, though it’s protection against the infections that we are analyzing here is very short in duration and likely less impactful. We do include both political leaning and insurance coverage, both of which are highly correlated with vaccination rates.

Major:

1) *(Observational analysis) I think the use of wavelets is a nice idea to tackle the multiple waves. I think Fig. 1A is fairly striking, however, I don’t find Fig. 1B/C (the county-level analysis) as convincing. There appears to be a lot of hetereogeneity at the county-level that is not driven by climate. For instance, why is there lower annual periodicity in the north west? What is it about the northwest that means we should expect similar periodicity to the south east (aren’t these regions “opposite” climat*e*wise?).*

We are glad that the reviewer liked our use of wavelet analysis. There is a lot of heterogeneity in the results, yet the correlations and R values are clear. Despite that heterogeneity, minimum temperature explains over a quarter of the variation in annual periodicity, which we consider a remarkably large amount for such an indirect correlate. While the Northwest and Southeast may appear to have “opposite” climates they both have warm winters relative to the rest of the country (as can be seen in Figure S5a), the climactic variable we have shown to be most correlated to annual periodicity. Based on minimum temperature, we would therefore expect both the Northwest and Southeast to have relatively little annual periodicity, which is also what we found (Figure 2c).

a) *Isn’t the value of wavelet analysis that the power spectra vary over time? But the authors aren’t using the temporal component in this analysis, so how does this differ from using a fourier/ lomb-scargle periodogram? Can you use the temporal component to say something more nuanced about how the periodicity has evolved and what the drivers might be?*

The value of wavelet analysis is that it can vary over time, and we recognize our oversight in not discussing this in detail. We have added an example wavelet and a discussion of how stable these wavelets are. We feel this is a very surprising and important result and are glad to have highlighted it in the revision.

b) *Why didn’t the authors consider specific humidity, given this is supported as a key driver of respiratory pathogen transmission (e.g. Shaman 2009,2010)?*

We agree that specific humidity would be an important variable to consider. We chose to instead focus on winter temperature to connect it other covid literature that looked at temperature and transmission (the reviewer’s references are about influenza) and practically because NOAA reports temperature already collated at a state and county scale but only reports humidity readings from specific weather stations. We would also like to note that winter temperature and specific humidity are highly correlated, and the results would likely be similar.

c) *The regression analysis (Table S1) is limited e.g. it’s strange to control for min and max temperature in the same regression model as they are so strongly correlated. I think the authors need to show that the estimated effect of temperature is robust to different specifications and controls.*

We agree that minimum and maximum are highly correlated, yet it is thus perhaps more surprising that they both are significant in the multivariable model. We also ran many more multivariable analyses that we did not include in the manuscript, including variable selection analysis. We felt these analyses were complicated to explain and added little to the discussion.

d) *The authors need to include climate data for Alaska and Hawaii etc. There are so many gridded global climate datasets out there that ignoring certain locations because you didn’t download the right climate data comes across as careless (ERA5, for example, is the gold standard).*

We agree that including climate data for Alaska and Hawaii (and Puerto Rico) would be ideal. We chose the NOAA dataset because it was already collated at the state and county level, rather than reported as grids or weather stations. As there are many ways to average a temperature time series over all the weather stations or grids in a county or state, we felt it was more prudent to lean on NOAA’s expertise and use their averages instead.

2) *(Model) I like the innovation in using the more sensitive formulation of waning/ partial immunity than might be provided by a basic SIRS model. Can the authors hint at any mechanistic reason, specific to COVID19, to using their model for this question versus a standard SIRS? There is some basic critique of SIRS on page 6 paragraph 2, but why does the SIRS, in not considering “partial or waning immunity but absolute immunity” matter for modeling COVID19?*

We are glad the reviewer liked the premise of our model. We added a few sentences on the motivation for our modeling decisions and why a classical SIRS is likely improper for this scenario.

a) *I think the author could do more to tie the modeling results from Fig 2 to the observations in Fig 1. Great that the model finds two outbreaks a year, but how does the model capture the seasonal gradient observed in Fig 1 i.e. why do northern locations have a stronger annual periodicity?*

We added some discussion more explicitly tying the modeling results to the climatic correlations and periodicity we found in the case-based analysis.

b) *In general I felt the modeling results could be more clearly compared with the observational findings.*

Same as above.

3) *Vaccination is completely missing from both the model and observational analysis. Surely vaccination will determine spatial heterogeneity in outbreak patterns? I think the authors need to consider this much more closely, including vaccination rates and boosters as possible determinants in their statistical model. I also think the authors need to consider vaccination in the mechanistic disease model – can we rule out vaccination as a determinant of seasonality?*

We note that vaccination is highly correlated with both insurance coverage and political leaning. We also agree that including vaccination in a model with seasonal forcing is a very interesting idea and welcome a follow-up investigation doing so, but regret that it is out of the scope of this work. We would just note that vaccination occurs at an annual (or likely longer) period and is thus unlikely to cause sub-annual dynamics, and that it is hard to imagine how vaccination rates would correlate with the temperature-correlated gradient we described.

4) *I think the authors could have done a better job at more deeply considering pathogen evolution. The authors attempt to discuss this broadly on page 8/9 but I think a quantitative analysis would have been more satisfying. It seems the authors are implying that the waning immunity in their model is not driven by evolution (paragraph 3 page 8) but could be driven by evolution (paragraph 2 page 9). Given the rich availability of strain data for SARS-CoV-2, and existing secondary analyses, can the authors more firmly rule out whether evolution is happening at a rate that could drive these sub-annual periodicities. Does it make sense that natural immunity wanes so quickly otherwise?*

We agree that a longer and more detailed discussion of evolution is warranted. As noted in our response to Reviewer #1, we added discussion of evolution and VOCs throughout the paper.

Minor

1) *Please use line numbers – it helps with review.*

We are happy to include line numbers if allowed, but we didn’t based on our understanding of the Science magazine LaTeX guidelines.

2) *The first paragraph is largely uncited which seems a little strange. I think some of these sentences should be supported by the literature? Is there not a review paper on seasonality and respiratory pathogens that could support “agents that transmit via the respiratory route are typically associated with a peak in the winter months”. I quite like Moriyama 2020 (ref below).*

Thank you for the suggestion. It is a very nice paper and the reference was added.

3) *Paragraph 2 seems unfinished. I wanted another sentence “However, as susceptibility declines, it might expected that…”*

We have decided paragraph 2 added little to the introduction and it was completely removed.

4) *Paragraph 3 – I thought the two peaks a year has been particularly clear overthe last two years as opposed to in the early pandemic (but I might be wrong). Can we say anything on how this seasonality appears to have evolved over time? Also, might be helpful to show the time series in the US from 2019 to the present in the supplement.*

We agree that we did not sufficiently discuss how the periodic dynamics change or don’t change over the pandemic. A figure and paragraph with these results were added.

5) *Why minimum temperature? Can you show the correlation with max and mean too. Is it less strong? Is there some mechanistic reason to focus on minimum temperature?*

We feel that we have already adequately explained why we focused on minimum temperature and results for maximum temperature were already reported as well.

6) *Fig 1: - sub-labels are not in plot (i.e. A/B/C). Would also be helpful to refer to subfigures in the text (i.e. which part of Fig1 should I be looking at ?).*

We are sorry for the omission. Sub-labels were added to the figures and references.

a. *Include a color bar on Fig. 1A (what does purple mean?)*

We added a color bar to Figure 1C.

b. *Caption should specify what the line is in B), what is the significance test, what are the p-values.*

The regression line and its confidence intervals were added to the caption.

c. *Page 7, last paragraph, missing parenthesis.*

Thank you, the parenthesis was added.

**Reviewer #3**

*I really liked the topic of this paper. We're going to put a lot of effort into understanding SARS-CoV-2 seasonality over the next 5 years, and this contribution is a great start. Comparing the different seasonal drivers of influenza and SARSCoV-2 seasonality is also going to be interesting, and the authors touch on this topic in their manuscript. The question is great, the approach is great, but I think the authors know that the data aren't there yet to answer this question. For now, I would submit this as an exploratory analysis to a traditional epidemiology journals. In order of importance, my comments, critiques, and suggestions are below:*

We are pleased that Reviewer 3 thought highly of our chosen topic and agree that the seasonality of COVID-19 is a very important and (mostly) yet to be discussed topic. We very much disagree with the assertion that the data are not yet available. Further discussion of our review is included in our specific response below.

1) *The SARS-CoV-2 incidence time series is simply too short to answer questions on COVID seasonality. 38 months and five peaks isn't enough, especially since we know many of the reasons for the occurrences of these individual peaks. Wave 1 ended because of the lockdowns in March-April 2020 (not because of any seasonal or waning factors). Wave 2 ended in Jan/Feb 2021 probably due to a combination of factors (lockdowns, fear) but definitely not because of population immunity. Wave 1 began because a new virus was introduced. Wave 2 began probably due to the seasonal and crowding factors mentioned in the paper. Wave 3 began becase the Delta variant arrived (not because immunity had waned). Wave 4 began because the somewhat immune-escaped and more trasmissible Omicron variant arrived (it would be very difficult to make the case for waned immunity here, but perhaps you could make the case for seasonal factors). I don't know what caused the summer 2022 wave (wave 5) and maybe this is the first wave where your analysis can begin. For waves 1 through 4, the other causal factors are simply too strong and you have no way of estimating the effects of seasonality or waning immunity for these four waves.*

We strongly disagree that the time series is too short. For one, we found little change in the periodicity over this period (new Fig 1) and reported striking correlations with climate over the time span studied. We strongly disagree that we “know many of the reason for the occurrences of these individual peaks”. As stated above, part of the motivation for this work is to examine factors beyond those reasons that have been assumed to underly the dynamics. While post-hoc explanations for each wave are readily available, we find regular timing of waves despite the arrival of VoCs at different times of the year. If VoCs were the dominant driver in wave dynamics, we would instead expect a much less regular pattern of periodicity. It is uncontroversial that seasonal forcing is important for influenza, rather than the specific influenza variant that was dominant at the time While influenza evolution can produce more severe flu seasons – especially if the result is a poor match to available vaccines – it does not alter the evident seasonality. We feel the attention that the Covid variants received from both the public and the scientific community should not prevent us testing for factors that are known to be important with similar pathogens.

2) *"Little long-term immunity" and "immunity wan[ing] relatively sharply" (bottom p7) are indeed consistent with sustained sub-annual periodicity. Very good point and it needs to be made. However, this is not consistent with the COVID burden and COVID epidemics we have seen the past two winters. The mild COVID epidemics of winter 2022-2023 and 2023-2024 suggest that immunity is in fact a bit longer than we initially thought, probably lambda > 700 days and not lambda < 200 days. References 21-23 are not very good references to support the lack of long-term immunity you are talking about. The Goldberg analysis is a very rich data set and an excellent analysis, but it only compares inside the range of 6-8 months of immunity to 0-2 months of immunity; you need to show that 10-14 months of immunity and 20-24 months of immunity is the same as 6-8 months, but these data don't exist (to my knowledge, I may not be up to date). The Hernandez-Suarez paper has better data, out to 14 months. But 14 months of 'some immmunity' in this paper cannot be used to justify a lamdba value <250 where almost all immunity is lost in 8 months. The Townsend PNAS paper gets has a pretty unusual approach to this question, but even if we take the results on face value, their results show 5% chance of reinfection 144 days after natural infection.*

We are glad Reviewer 3 agrees with our focus on waning immunity. As they note, estimating the waning functions from observational data is an incredibly difficult task. We cite these papers as references for the general shape of the waning function and a general notion of the parameter regime. Our model is relatively simple, though it can produce complex dynamics, and is based on few parameters. Our work here was not to fit it to specific estimates of immunity but rather to show that specific shapes of immune function (i.e., those that wane sharply and with little-long term immunity) can be a sufficient explanation for sub-annual periodicity. We welcome follow-up modeling efforts that attempt to create a more biologically motivated model, and one fitted to biological estimates of immunity, but that is both outside the scope and intent of this paper.

3) *Wavelet approaches result in a lot of overfitting to short-term epidemic time series. My guess is that the authors are aware of this. The Grenfell paper (ref 6) has 40+ years of data and a clear non-stationary pattern, but if you use a wavelet approach on 38 months of data, a wavelet basis function can easily shift its amplitude to fit a large peak -- its period will change as well when this happens -- and this can result in a particular periodicity being inferred. Also, what are the black lines in Figure S1? The regions of high confidence?*

We disagree that wavelets lead to overfitting. The first omicron wave was inarguably a large peak, yet the wavelets change little, if at all around that time. We included an example wavelet and a more detailed discussion of how they change over time that will hopefully relieve these concerns. Explanations for the black lines were added to the figure captions, and we apologize for the oversight.

4) *Figures 3 and S9 are really great, and I was surprised you could get conclusions in some of these analyses. This is an excellent diagnostic approach to looking for something rare in epidemiology (here, sub-annual periodicity).*

We are grateful that the simulation results were appreciated. As noted, this type of result is unusual in epidemiological modeling, and we feel they are an important contribution to the discussion of epidemic waves.

5) *Bottom of page 7, after you introduce all the parameter detail, it feels like you should be able to fit your model to the data here with a likelihood approach.*

We agree that we could use likelihood approaches to fit our model, but as we noted in our response to Reviewer 1, we feel that fitting a simple mechanistic model such as this is not a very useful practice. Our model was introduced as a relatively simple way to test different forcing and waning scenarios, not to be a precise forecast .

6) *Authors say "here we show short-term immunity that wanes quickly is a sufficient condition for regular sub-annual periodicity, independent of multi-strain dynamics". Yes, true. Thieme and Yang (2002, Math Biosci 180:207) is the basis for this.*

Thank you for the reference to Thieme and Yang. We added a reference to their work.

7) *There must be some evidence in the sub-tropical and tropical influenza literatureon sub-annual periodicity. Hong Kong has 20+ years of data and biannual peaks. Some older papers (Deyle PNAS 2016, Tamerius 2013 PLoS Pathog) have good summaries of this, but I don't know if they explicitly look at sub-annual periodicities.*

While the papers listed here by Reviewer 3 are fantastic works, they show that influenza dynamics in the tropics are not annual, but they don’t establish a regular sub-annual periodicity to their epidemics.

8) *For influenza virus, we know a little bit more about environmental drivers than you let on. The best mechanistic descriptions are in Wan Yang's papers on droplet size and the U-shaped absolute humidity relationship for influenza transmission.*

There is extensive literature on the mechanistic drivers of influenza seasonality. We chose to only mention it in passing to not distract from our focus on covid-19.

9) *It is plausible that the northern states have more pronounced winter peaks; this is also true in flu data.*

We mention that this is indeed the case.

10) *I wouldn't use "tropical climates" in your ms; maybe "counties closer to subtropical zones".*

Thank you for the suggestion. We removed any mentions of topical and temperate climates and instead focused on the more accurate temperatures.

11) *The winter peak being close to the holiday period: there is no way to determine if this is a coincidence or not. Flu peaks are only sometimes close to the holiday period.*

We agree that the holiday period is likely confounding and difficult to disentangle. We mention this in our discussion but still think the changes in transmission networks around that time are likely a source of many infections and important to note.

12) *I don't understand what the dose-response relationship is on page 5.*

Sorry for the confusion. We updated the language to convey our meaning more clearly.

13) *Introduction, para 1: COVID waves can also be caused by varying patterns of response to lockdowns and the arrival of new variants.*

We added a mention to lockdowns and welcome a follow-up study investigating mitigation measures and periodicity. We would just note that the political affiliation has little to no effect on the periodic dynamics.
