# Supplementary material for: Seasonal forcing and waning immunity drive the sub-annual periodicity of the COVID-19 epidemic
Source: PLoS Pathog. 2026 Apr 27;22(4):e1014169. doi: 10.1371/journal.ppat.1014169 (PMC13138748; doi:10.1371/journal.ppat.1014169)
Supplement: Attachment — Submitted filename: Response_to_Reviewer.docx [file ppat.1014169.s018.docx]

Response to the Reviewer

We would first like to thank the reviewer for their careful reading and review of our manuscript. We are very pleased they found our last round of edits an improvement and that they see the significance of the work. We were happy to take their advice for every comment they left. A specific account of the comments is below.

Major issues:

**Model equations (1-5) in the Results section duplicate model equations (9-12) in the Methods section. I suggest removing equations (1-5) and keeping equations (9-12) in the Methods section since the model provides a Method for analysis.**

We agree that removing the model details in the Results is a duplication and unnecessary. We have removed them.

**It would be helpful to get an idea of what the annual attack rates are of the subset of simulations that reproduce characteristics of the observed seasonal patterns (e.g., attack rates of simulations using immunity waning functions shown in Fig S7). Are these annual attack rates reasonable/supported by empirical data, e.g., serological surveys/longitudinal studies of antibody dynamics? With such rapid rates of waning and the dynamics shown in the first row of Fig S7, my guess would be that annual attack rates would be relatively high? (Greater than the 10-20% estimated for flu.)**

Understanding the attack rates of the different parameterizations of our model is a really interesting question. We have added a figure in the Supplement to show how attack rates change as a function of immunity half-life and a brief discussion of attack rates in the main text.

In general, our model has a very simple transmission mechanism as it is an SIRS model with more nuanced immunity, but no change to the transmission or infection terms (we left R_0=2.5 and the infectious period = 7 days fixed for all simulations). Such SIRS style models are the foundation of much of our understanding and intuition about infectious disease dynamics, but a common feature is that they generate (damped) waves that oscillate around an endemic mean number of infections and do not return to a very low or close to zero number of infections in the off-season between peaks without additional elements. This oscillatory behavior can result in some unrealistically high attack rates. While these sorts of dynamics are well known (e.g., discussed in both Anderson and May’s and Vynnycky and White’s textbooks), they mean that such models are better suited to discussing qualitative or comparative results such as expected seasonality, than specific comparisons to real-world statistics. That being said the main qualitative finding from comparing attack rates is that they are largely determined by the number of waves per year as the disease runs through the population (with per wave attack rates between 50-100%) each wave. Simulations with very (unrealistically) short immunity half-lives can result in 5+ waves per year and therefore similarly high attack rates. Simulations parameterized with longer and more biologically plausible durations of immunity have much lower attack rates close to or below 1.

In comparison to flu, COVID-19 has a much shorter duration of immunity (on the scale of months rather than a few years) and we would therefore expect it to have higher annual attack rates as multiple waves of the virus are taking place each year, even if these are largely made up of mild infections that may not be immediately apparent.

**At the state level, how were the min and max temperatures calculated? Were measurements from the most highly populated county used or data somehow averaged across counties (weighted by population size)? Lines 450-452 do not provide sufficient information on this.**

We are sorry this was not specified. All of the aggregation of the climate data was handled by NOAA as county and state-level averages are already included in their nClimGrid datasets. These regional averages are latitude weighted averages of all of the points within each region and do not weight by population. This information was added to the methods section.

Minor issues:

**Abstract: please mention time period of COVID studied (the author summary indicates ‘during the pandemic period’ but this is not mentioned in the abstract)**

It has been added.

**Line 14 (“In the case of the COVID-19 epidemic…”): please include some citations here. If a reader 10 years down the line is reading this paper, these waves might not be in their working memory**

A citation has been added.

**Line 47: 7-day rolling average: this is sensible but perhaps include an explanation of why you chose 7-days for the rolling average. (Are there day-of-the-week reporting trends?)**

We have added an explanation for this decision. In short, daily cases data was very messy and chaotically collected during the pandemic period. While not shown or discussed, we also did run all of the analyses on the raw daily case data and there were no real changes to the results.

**End of Intro: county-level analysis is indicated but large portion of the Results are at the state level. This seems inconsistent. I suggest adding text to the end of the intro section indicating that both state and county level analyses will be performed.**

It has been added.

**Fig 2B: The linear regression does not seem to capture the data points very well. Consider (only for this panel) to also show a spline fit or logistic-like curve to highlight that there might be a threshold effect?**

We agree that the linear regression does not completely capture the nature of this relationship. Our goal was simply to show that warmer winters can lead to more subannual periodicity. We have added a version of the figure with a spline fit into the Supplement and would very much welcome follow-up work to further tease apart the relationship between weather and epidemic seasonality.
